# Supplementary material for: Causal Relationship between Aging and Anorexia Nervosa: A White-Matter-Microstructure-Mediated Mendelian Randomization Analysis
Source: Biomedicines. 2024 Aug 16;12(8):1874. doi: 10.3390/biomedicines12081874 (PMC11351342; doi:10.3390/biomedicines12081874)
Supplement: Supplementary file 1 [file biomedicines-12-01874-s001.zip › Supplementary Figure/Figure S4/Singe SNP plot of MR analysis for TL to ubm-b-1862.pdf]

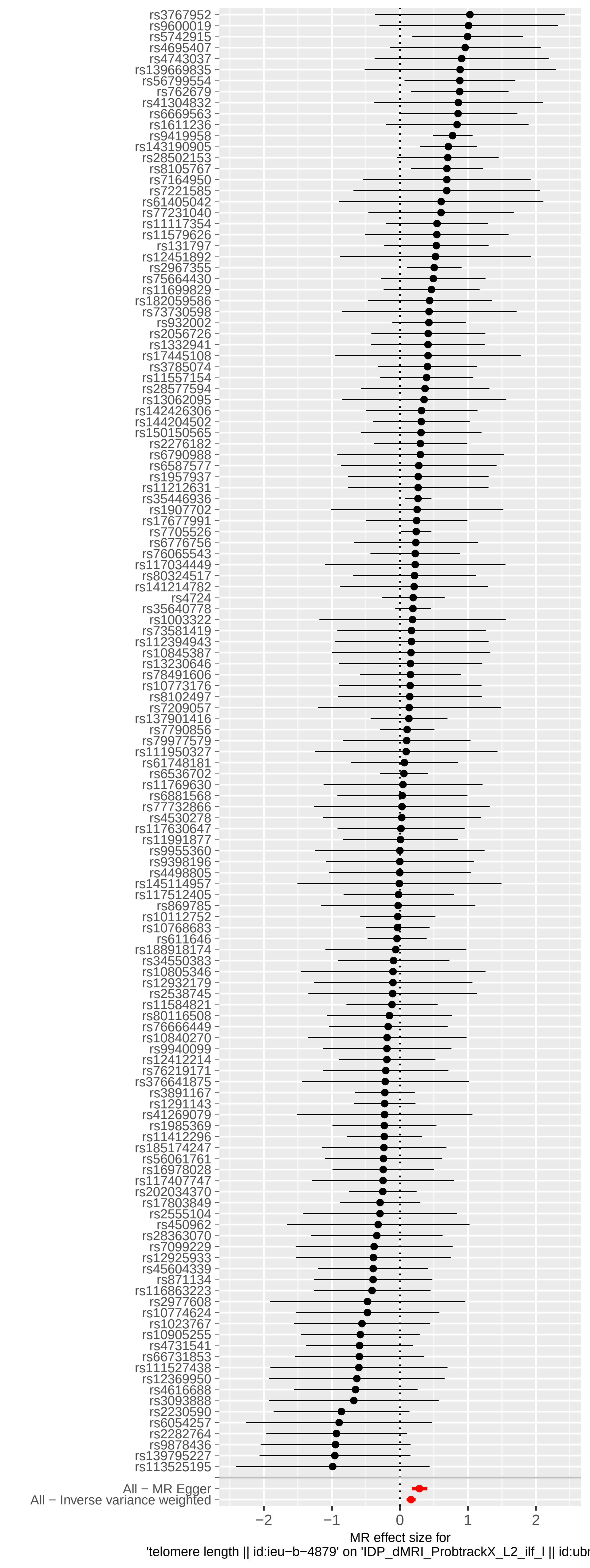

rs3767952
rs9600019
rs5742915
rs4695407
rs4743037
rs139669835
rs56799554
rs762679
rs41304832
rs6669563
rs1611236
rs9419958
rs143190905
rs28502153
rs8105767
rs7164950
rs7221585
rs61405042
rs77231040
rs11117354
rs11579626
rs131797
rs12451892
rs2967355
rs75664430
rs11699829
rs182059586
rs73730598
rs932002
rs2056726
rs1332941
rs17445108
rs3785074
rs11557154
rs28577594
rs13062095
rs142426306
rs144204502
rs150150565
rs2276182
rs6790988
rs6587577
rs1957937
rs11212631
rs35446936
rs1907702
rs17677991
rs7705526
rs6776756
rs76065543
rs117034449
rs80324517
rs141214782
rs4724
rs35640778
rs1003322
rs73581419
rs112394943
rs10845387
rs13230646
rs78491606
rs10773176
rs8102497
rs7209057
rs137901416
rs7790856
rs79977579
rs111950327
rs61748181
rs6536702
rs11769630
rs6881568
rs77732866
rs4530278
rs117630647
rs11991877
rs9955360
rs9398196
rs4498805
rs145114957
rs117512405
rs869785
rs10112752
rs10768683
rs611646
rs188918174
rs34550383
rs10805346
rs12932179
rs2538745
rs11584821
rs80116508
rs76666449
rs10840270
rs9940099
rs12412214
rs76219171
rs376641875
rs3891167
rs1291143
rs41269079
rs1985369
rs11412296
rs185174247
rs56061761
rs16978028
rs117407747
rs202034370
rs17803849
rs2555104
rs450962
rs28363070
rs7099229
rs12925933
rs45604339
rs871134
rs116863223
rs2977608
rs10774624
rs1023767
rs10905255
rs4731541
rs66731853
rs111527438
rs12369950
rs4616688
rs3093888
rs2230590
rs6054257
rs2282764
rs9878436
rs139795227
rs113525195
All − MR Egger
All − Inverse variance weighted
−2
−1
0
1
2
MR effect size for
'telomere length || id:ieu−b−4879' on 'IDP_dMRI_ProbtrackX_L2_ilf_l || id:ubr
